# Pheochromocytoma Leading to Multiorgan Failure in a Pregnant Patient: A Case Report

**DOI:** 10.5811/cpcem.2021.6.52727

**Published:** 2021-08-31

**Authors:** Toby Myatt, Margot Barker

**Affiliations:** University of California, Irvine, Department of Emergency Medicine, Irvine, California

**Keywords:** medicine, pheochromocytoma, pregnancy, case report

## Abstract

**Introduction:**

Pheochromocytoma, a neuroendocrine tumor that secretes catecholamines, can present with episodic sweating, diaphoresis, headaches, and hypertension, as well as cardiac and pulmonary involvement. In a pregnant patient, it must be differentiated from preeclampsia, a leading cause of maternal mortality in the developed world, which can similarly present with hypertension and multiorgan involvement. Both conditions require early diagnosis and treatment to reduce maternal and fetal morbidity and mortality.

**Case Report:**

We discuss the case of a pregnant patient at approximately 24 weeks’ gestation presenting with chest pain and shortness of breath who was found to have a left adrenal mass and hypertensive urgency. The patient acutely decompensated during the course of evaluation. She ultimately suffered pregnancy loss and multiorgan failure requiring percutaneous heart pump placement and extracorporeal membrane oxygenation therapy for support before fully recovering. The adrenal mass was confirmed to be a pheochromocytoma after excision and contributed to the development of hypertensive emergency with multiorgan failure.

**Conclusion:**

Pheochromocytoma during pregnancy is a rare condition but must remain on the differential until ruled out to improve patient outcomes as much as possible. Obtaining blood pressure control is imperative to reducing maternal and fetal mortality. Preeclampsia is similarly serious, and early diagnosis is essential for adequate management of the condition until delivery can occur.

## INTRODUCTION

Pheochromocytoma is a neuroendocrine tumor that secretes catecholamines, derived from chromaffin cells of the adrenal medulla or extra-adrenal paraganglia.^1^ Typically, patients present with episodic sweating, diaphoresis, headaches, and hypertension, although presentations can be varied and multiple organ systems can be involved.^1^,^2^ Cardiovascular and pulmonary involvement can also be associated with pheochromocytoma, including coronary spasm, arrhythmias, myocardial infarction, hemodynamic collapse, heart failure, pulmonary edema, and even cardiac arrest.^3^–^5^ Diagnosis requires biochemical confirmation with urine or plasma fractionated metanephrines.^6^ This is followed by radiologic studies to locate the tumor and resect if anatomically feasible.^6^ It must be differentiated from preeclampsia, a condition with a very different etiology, that can also lead to multiorgan system involvement and is a leading cause of maternal mortality in the developed world.^7^ It is defined as new-onset hypertension after 20 weeks’ gestation with evidence of maternal organ dysfunction, proteinuria, or uteroplacental dysfunction.^8^ Early diagnosis and treatment with a multidisciplinary approach are imperative, particularly in the case of a pregnant patient.

## CASE REPORT

**Image 1:**
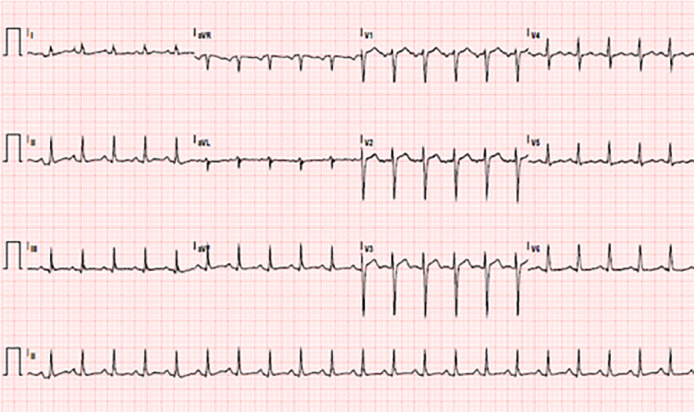
Electrocardiogram showing sinus tachycardia at 129 beats per minute.

A 36-year-old pregnant female (self-reported 24 weeks and 1 day gestation, with history of two prior pregnancies resulting in one pre-term delivery and one full-term delivery) with a history of type 2 diabetes mellitus presented to the emergency department (ED) with a history of unprovoked, pressure-like chest pain for 3.5 hours. It was associated with shortness of breath, cough and nausea, and was worse when lying down. The patient had discovered she was pregnant two weeks previously and denied prior prenatal care or ultrasound. Additionally, she had recently been diagnosed with hypertension and started taking 100 milligrams (mg) labetalol twice daily one day before presenting. She had not taken labetalol on the day of her admission. In the ED, the patient was found to be severely hypertensive with systolic blood pressures (BP) in the 180s millimeters of mercury (mm Hg) and diastolic BP in the 120s mm Hg. She was also hypoxic with peripheral capillary oxygen saturation in the mid-80s percent on room air. An electrocardiogram (ECG) revealed sinus tachycardia at 129 beats per minute (Image 1).

Initial differential diagnosis included preeclampsia due to the recent diagnosis of hypertension, as well as pulmonary embolism, peripartum cardiomyopathy, and cardiac ischemia. The patient continued to experience worsening dyspnea and hypoxia despite trials on non-rebreather and bilevel positive airway pressure, and she was intubated. She was given 40 milligrams (mg) intravenous (IV) furosemide due to concern for flash pulmonary edema. She was then started on IV nitroglycerin and nicardipine as her BP remained consistently elevated to greater than 200/110 mm Hg. Laboratory studies revealed a pH of 6.9 (reference range: 7.35–7.45); bicarbonate of 11 milliequivalents per liter (mEq/L) (22–28 mEq/L); potassium of 7.9 mEq/L (3.5–5.0 mEq/L); and cardiac troponin that trended upward from 0.14 nanograms per milliliter (ng/mL) to 1.4 ng/mL (reference range: less than 0.04 ng/mL). Plasma-free normetanephrine was elevated at 273 nanomoles per liter (nmol/L) (reference range: less than 0.9 nmol/L), and free metanephrine was elevated at 4.8 nmol/L (less than 0.5 nmol/L).

**CPC-EM Capsule**

What do we already know about this clinical entity?

*Pheochromocytoma is a rare condition that can present with varied symptoms and multisystem involvement that can present severely in the emergent setting.*

What makes this presentation of disease reportable?

*Early diagnosis and treatment of pheochromocytoma is necessary to reduce maternal and fetal morbidity and mortality.*

What is the major learning point?

*Reinforces the importance of a broad and comprehensive differential when evaluating patients in the emergency setting.*

How might this improve emergency medicine practice?

*By adding to the body of knowledge regarding diagnosis and management of pregnant patients presenting with chest pain and shortness of breath in the emergency setting.*

**Image 2:**
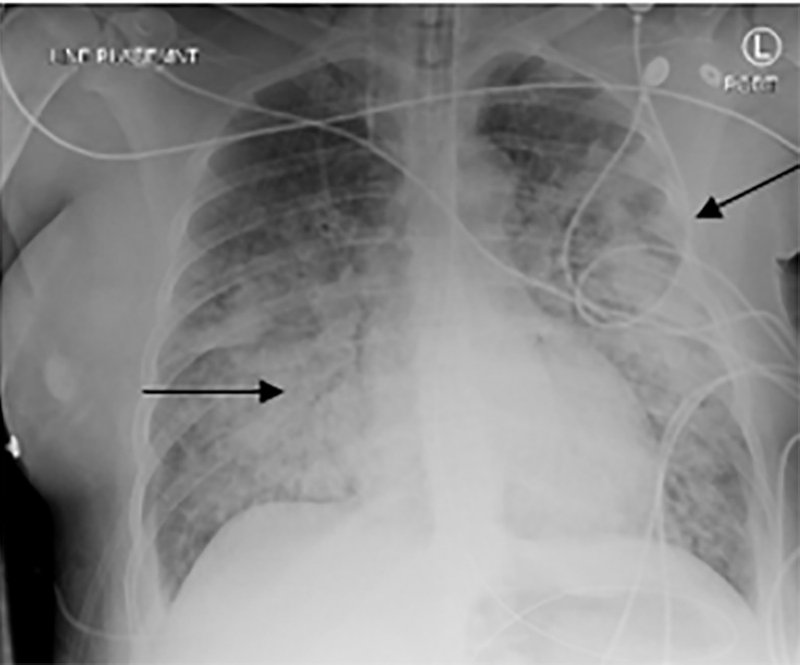
Chest radiograph of patient with arrows showing diffuse pulmonary edema

**Image 3:**
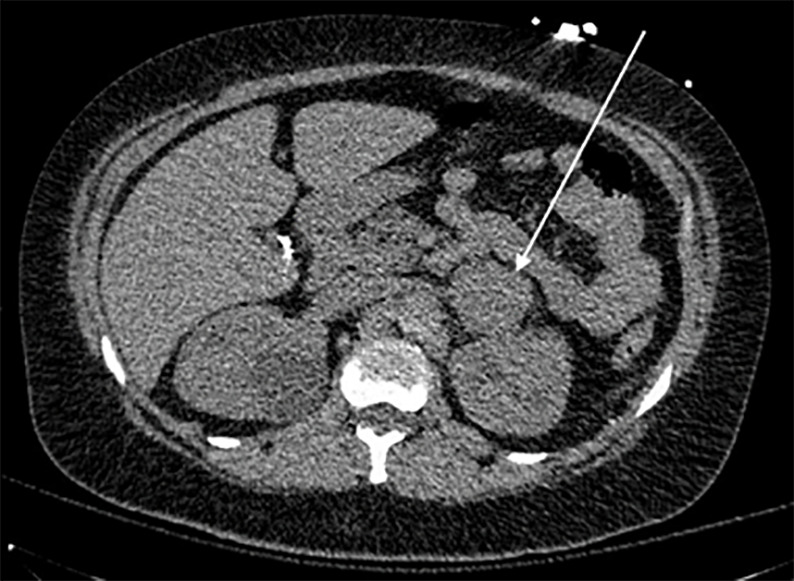
Computed tomography showing soft tissue density lesion measuring approximately 4 centimeters (cm) × 4.5 cm in the left suprarenal region.

Point-of-care ultrasound (POCUS) revealed B-lines consistent with severe pulmonary edema, an enlarged heart with depressed ejection fraction, and a live intrauterine pregnancy with fetal heart tones in the 120s–140s beats per minute. Computed tomography (CT) angiography of the chest was negative for pulmonary embolism. Both CT and chest radiograph showed diffuse pulmonary edema (Image 2). Computed tomography also revealed a suprarenal mass (Image 3). A repeat POCUS performed on day of presentation revealed intrauterine fetal demise.

The patient was given bicarbonate and insulin and glucose for profound acidosis and hyperkalemia, and she was transferred to the medical intensive care unit (MICU). She then experienced several convulsive episodes, which were initially thought to be eclamptic in etiology. She was first treated with 4 mg IV lorazepam rather than magnesium due to concern for potential exacerbation of pulmonary edema, but eventually required 2 mg magnesium to resolve seizures. The patient then became hypotensive and hypoglycemic and was treated with two ampules of 50% dextrose in water and started on vasopressors before undergoing emergent, uncomplicated cesarean section due to the presence of a large lower segment/cervical fibroid blocking access from the cervix.

The patient was returned to the MICU where she remained persistently hypotensive on milrinone, dobutamine, epinephrine, vasopressin, and norepinephrine. She was intermittently in ventricular tachyarrhythmia, with associated worsening of hypotension. A repeat ECG showed peaked T-waves with right bundle branch block and widened QRS complexes, suggestive of hyperkalemia. The ECG changes resolved with administration of calcium chloride. Nephrology recommended initiation of continuous renal replacement therapy, which improved her hyperkalemia and resolved her ventricular tachyarrhythmia. However, the patient continued to be hypotensive despite maximal vasopressor support. A formal echocardiogram revealed an ejection fraction of 15%, prompting placement of a ventricular assist device for circulatory support.

At this point, the patient was suffering from multiorgan failure including renal failure, shock liver, acute heart failure, respiratory failure, and disseminated intravascular coagulation. She was still requiring the maximum dose of five vasopressors at that time. Her blood pressure remained stable until the next morning, when her mean arterial pressure (MAP) dropped to the 40s mm Hg. Continuous renal replacement therapy was stopped with improvement in her MAP improved to the 60s mm Hg. The patient was transferred to an outside facility for extracorporeal membrane oxygenation therapy. She was stabilized after several days and tolerated removal of the ventricular assist device. After continued supportive therapy, her organ function began to improve, with recovery of her heart, lung, liver, and kidney function. The patient subsequently underwent excision of the adrenal mass, which was confirmed to be a pheochromocytoma.

## DISCUSSION

Pheochromocytoma can be difficult to diagnose, as it can present with varied symptoms and lead to multisystem organ involvement. However, it is imperative to consider because it can lead to severe cardiovascular complications because of excessive catecholamine release and can be fatal if left untreated.^3^,^4^,^9^ Preeclampsia is a condition that must be treated with a similar level of caution. While its etiology is unclear, it is theorized that it develops due to defective spiral artery formation and remodeling, which then leads to cellular ischemia in the placenta.^10^,^11^ This causes a release of pro-inflammatory, anti-angiogenic factors, leading to widespread endothelial dysfunction affecting all maternal organ systems.^10^,^11^ These alterations in vascular function contribute to the development of hypertension as well as multiorgan dysfunction, which is more severe with early-onset preeclampsia.^10^,^12^ Additionally, women with diabetes and women with chronic hypertension are more likely to develop preeclampsia, both risk factors that were present in our patient.^8^,^13^

Early diagnosis and management of both conditions is essential to reducing patient morbidity and mortality. Our patient had no previous obstetric care, likely secondary to her lack of awareness of her pregnancy. This, coupled with her atypical and severe presentation, made diagnosis especially difficult. Suspicion was raised for pheochromocytoma due to the patient’s refractory hypertension and incidentally observed suprarenal mass on imaging, which prompted diagnostic confirmation with plasma metanephrines and eventual surgical pathology. In addition, there was significant cardiac involvement, as well as pulmonary edema and multiorgan dysfunction. Excessive catecholamine exposure, such as that caused by a pheochromocytoma, can cause cardiac dysfunction by inducing intracellular calcium overload in cardiomyocytes.^3^ This can cause a variety of pathological cardiac symptoms, including coronary spasm, arrhythmias, myocardial infarction, hemodynamic collapse, heart failure, pulmonary edema, and even cardiac arrest.^3^,^4^ Preeclampsia was also included in the differential due to her elevated BP on presentation, seizure activity, and significant reduction in BP following delivery.

Pheochromocytoma can be especially disastrous during pregnancy, occurring in approximately 1 in 54,000 pregnancies.^14^,^15^ In addition to hypertension, pheochromocytoma can cause altered renal function and proteinuria as a result of catecholamine-induced renovascular abnormalities.^15^ Because it is so rare, pheochromocytoma is often initially misdiagnosed as preeclampsia.^15^ Diagnosis requires biochemical confirmation of elevated catecholamine levels: either 24-hour urine fractionated metanephrines and catecholamines or plasma fractionated metanephrines followed by radiologic studies to locate the tumor.^6^ It is imperative to obtain BP control as soon as possible for all patients with pheochromocytoma, but especially in pregnant patients to prevent fetal demise, typically with alpha-blockers, beta-blockers, and magnesium sulfate.^15^ Fetal loss associated with maternal pheochromocytoma is reported to be 11%.^14^ Unfortunately, our patient presented with hypertensive crisis and fetal demise could not be prevented.

Definitive treatment of pheochromocytoma is surgical resection, and the timing during pregnancy is controversial.^15^ For first trimester cases, surgical resection is not recommended due to high rates of miscarriage, while adrenalectomy is typically performed for patients presenting in their second trimester.^15^ For patients presenting in the third trimester, surgical resection is often delayed until delivery and then performed concurrently with C-section.^14^,^15^ Medical management, performed until surgical intervention can occur, consists of alpha-blockers, beta-blockers, and magnesium sulfate.^15^ The alpha-blocker should be started before the beta-blocker to prevent unopposed alpha receptor stimulation.^15^

Magnesium sulfate is used in the treatment of both pheochromocytoma and preeclampsia because it reduces BP, inhibits catecholamine release, blocks peripheral catecholamine receptors, and causes vasodilation.^15^ Definitive treatment of preeclampsia is delivery of both the baby and the placenta.^8^ In the case of our patient, the fetus did not survive the patient’s hypertensive crisis, and surgical resection of the adrenal mass was deferred until after stabilization and discharge to an outside facility. Pheochromocytoma was later confirmed on surgical pathology. Although preeclampsia was initially on the differential, given the presence of the suprarenal mass and elevated catecholamines, the patient was diagnosed with pheochromocytoma leading to the development multiorgan failure.

Early diagnosis of both pheochromocytoma and preeclampsia, especially with atypical presentation, remains difficult. In this case, the patient’s shortness of breath and chest pain led to imaging that showed diffuse pulmonary edema and suprarenal mass. These findings, combined with her persistent hypertension, raised suspicion for both pheochromocytoma and preeclampsia. Unfortunately, pheochromocytoma often goes undiagnosed, especially when presenting with atypical symptoms. In patients without prenatal care, preeclampsia can also go undiagnosed until it becomes severely symptomatic. Given the life-threatening complications that can occur because of both conditions, diagnosis and multidisciplinary management are essential for reducing maternal and fetal mortality.

## CONCLUSION

Pheochromocytoma during pregnancy is a rare condition but must remain on the differential until ruled out to improve patient outcomes as much as possible. Obtaining blood pressure control is imperative to reducing maternal and fetal mortality. Preeclampsia is similarly serious and early diagnosis is essential for adequate management of the condition until delivery can occur.
